# A Comparative Analysis of Emulated and Real IEC-104 Spontaneous Traffic in Power System Networks

**DOI:** 10.1007/978-3-030-69781-5_14

**Published:** 2021-01-28

**Authors:** C.-Y. Lin, Simin Nadjm-Tehrani

**Affiliations:** 8 Norwegian Computing Center, Oslo, Norway; 9 University of Trento and Fondazione Bruno Kessler, Trento, Italy; 10 Università degli Studi di Genova, Genoa, Italy; 11 IEIIT Institute, Consiglio Nazionale delle Ricerche (CNR), Genoa, Italy; 12 SINTEF A.S., Oslo, Norway; 13 Engineering Ingegneria Informatica S.p.A., Rome, Italy; 14 Instituto Superior de Engenharia do Porto, Porto, Portugal; 15 University of Padua, Padua, Italy; Department of Computer and Information Science, Linköping University, Linköping, Sweden

**Keywords:** SCADA, Traffic characterization, IEC-104, Timing analysis

## Abstract

Supervisory and Data Acquisition (SCADA) systems control and monitor modern power networks. As attacks targeting SCADA systems are increasing, significant research is conducted to defend SCADA networks including variations of anomaly detection. Due to the sensitivity of real data, many defence mechanisms have been tested only in small testbeds or emulated traffic that were designed with assumptions on how SCADA systems behave. This work provides a timing characterization of IEC-104 spontaneous traffic and compares the results from emulated traffic and real traffic to verify if the network characteristics appearing in testbeds and emulated traffic coincide with real traffic. Among three verified characteristics, two of them appear in the real dataset but in a less regular way, and one does not appear in the collected real data. The insights from these observations are discussed in terms of presumed differences between emulated and real traffic and how those differences are generated.

## Introduction

A modern power distribution system is a cyber-physical system comprising a network of geographically distributed devices and processes. Supervisory Control and Data Acquisition (SCADA) systems are used to control and monitor the network and processes. The emergence of attacks targeting SCADA systems and the controlled processes makes SCADA security a pressing issue [6, 8, 18]. Research on defending SCADA networks against such intrusions requires traffic datasets to develop, evaluate, and compare different defence mechanisms. Due to the secrecy of SCADA systems as part of critical infrastructure, real traffic is not openly available for the research community. Where data sharing from a large testbed is available, for example, in the case of iTrust testbed data from the EPIC and SWaT testbeds [11], the packet flows have been generated from one of the many possible SCADA protocols (CIP, GOOSE, MMS). Unfortunately, many defence mechanisms that need to be tested with other protocols, e.g. Intrusion Detection Systems (IDS) for IEC-60780-5-104 and IEC-61850 protocols are tested on a small-scale testbed [24] or with simulated/emulated datasets [13, 25]. Hence, how reliable are the simulated/emulated datasets has become a crucial question for the development of SCADA-specific IDS.

IEC-60780-5-104 (hereafter referred to as IEC-104) protocol is an international standard of data transmission between an electric power SCADA center and outstations over TCP/IP and widely used in Europe [7]. Unlike other SCADA protocols, such as Modbus, comprising mainly request-response communications triggered by a polling mechanism from the SCADA center, IEC-104 traffic contains a great deal of spontaneous communications [21]. In the spontaneous communication mode, field devices in the outstations initiate messages when the monitored measurements of process variables change or fall outside a predefined range.

Most of the research on IDS for SCADA networks model the request-response communications [5, 12, 23, 25] but fewer solutions are available on spontaneous communications due to the lack of understanding of spontaneous traffic. Although Lin and Nadjm-Tehrani [19] applied pattern mining techniques based on Probabilistic Suffix Tree (PST) to two emulated IEC-104 datasets in order to discover timing patterns, study of IEC-104 traffic characteristics is still in its infancy. Detailed knowledge of how spontaneous traffic behaves in a real network is necessary for the development of SCADA-specific IDS and improved SCADA network simulations/emulations.

This work first reviews potential flow-based characteristics suggested in literature, and then provides an empirical study of spontaneous traffic generated in a real-world utility with respect to the reviewed characteristics. It also performs a comparison with the emulated traffic used in previous works [19, 24].

Our primary contribution in this study is a detailed timing-based characterization of IEC-104 spontaneous traffic collected from a real power station. The results can be a first step to arrive at a traffic model when deciding about features and modeling approaches for anomaly detection, expanding the possibility of testing those IDS so far only tested with simulated/emulated traffic. The secondary contribution is the outcome of the comparison between behaviour of traffic from real and emulated power networks. It suggests the emulated traffic generated in earlier works may not be realistic enough. Some modifications could be made in those testbeds to improve the usability of the datasets generated for SCADA security research.

## Related Work

To guide and facilitate intrusion detection research for SCADA systems, network analysis and characterization of SCADA traffic has been an active research area. Most of the works focus on characterizing high level attributes such as bandwidth, port number, and the number of protocols. Barbosa et al. [3] conducted a comparative analysis of SCADA traffic from water treatment facilities and normal IT traffic. This study found that SCADA traffic lacks traffic patterns that are used to model IT traffic. The results indicate the need for SCADA-specific modeling approaches for anomaly detection. In separate work, Barbosa et al. [4] conducted another comparative analysis of SCADA traffic and SNMP traffic and found that both of them exhibit periodicity. Jung et al. [14] characterized SCADA traffic of a power station with variations in frame sizes, TCP connects, port number, and initial sequence number. In a later work, Formby et al. [9] further studied the initial sequence number attribute in the same traffic and found predictable patterns.

As more intrusion detection studies focus on protocol-specific models, more protocol-specific attributes are explored. Formby et al. [10] characterized DNP3 power grid traffic and examined a few common hypotheses such as stable traffic volume and regularity of DNP3 poll time. Mai et al. [22] characterized IEC-104 power grid traffic regarding the number of occurrences of different instructions and the directions and magnitude of IEC-104 flows, where the flow is defined by the source and destination addresses with the 4-tuple $srcIP, srcPort, dstIP, dstPort$ . Lin and Nadjm-Tehrani [19] characterized emulated IEC-104 spontaneous traffic with a focus on the predictability of timing patterns.

The current paper examines three characteristics of IEC-104 spontaneous traffic using data collected from a real power station. Two of the characteristics were proposed or observed in earlier work [19]. One characteristic observed in the emulated datasets of the earlier work is confirmed when analysing the real traffic while another characteristic is shown to exist only in the emulated datasets. The third characteristic will be discussed in more detail in Sect. 5. The confirmed characteristics have already guided the development of an anomaly detector [20] for IEC-104 spontaneous traffic.

## Background on the IEC-60780-5-104 Protocol

The IEC-104 protocol is widely used in modern SCADA systems to control and monitor geographically dispersed processes, especially for power station automation. The main advantage of IEC-104 is that it connects a control station (Master Terminal Unit, MTU) and one or more substations (Remote Terminal Unit, RTU) via a standard TCP/IP network. The IEC-104 protocol defines two directions for data transmission: (1) monitor direction, the direction of transmission from an RTU to the MTU and (2) control direction, the direction of transmission from the MTU to an RTU. The monitored data that are transmitted from an RTU to the MTU are also known to be sourced at *Monitor Points*. Every monitor point is configured to locate in a specific address in an RTU device and will be identified by the address at application level.

In order to improve the communication efficiency, the IEC-104 protocol enables not only the MTU to poll for monitor points periodically but also the RTUs to generate spontaneous events about data changes at monitor points. The following explains the important terminologies of IEC-104 protocols used in this study.

- Information Object: A piece of data containing information from a monitor point such as measured value and time tag. A spontaneous packet may carry more than one information object.
- Information Object Address (IOA): The address and identification of a monitor point where an information object is issued from.
- Cause of Transmission (COT): A field in the application layer to specify the type of the packet. A spontaneous packet is noted as SPONT.
- Type IDentification (TID): A field in the application layer to specify the type of the monitor point(s) in a packet. The most common data type is Monitored MEasured point in different formats such as M_ME_NA (normalized value) and M_ME_NB (scaled value). This data type contains a measured value from a certain IOA. The system administrator needs to set a deadband (i.e., a range) for each monitored measured point and the RTU will send a spontaneous event when the value falls outside the deadband. In addition, Monitored Single Point (e.g., M_SP_NA) and Monitored Double Point (e.g., M_DP_NA) specifies the state of a point, such as a switch or circuit breaker. For these points, the RTU will send a spontaneous event whenever the value changes.

## The Studied Datasets

This section first presents an overview of the examined datasets. Then, it describes how the datasets are collected and preprocessed for the analysis.

In this study we analyze three different IEC-104 datasets: two emulated power network datasets and one dataset collected in a real power station at a utility.

- **SmallTB-RTUx:** SmallTB-RTUx dataset is collected from a small testbed with real commercial hardware maintained by the Royal Institute of Technology (KTH). The setup contains four RTUs, one switch, and a user terminal machine. The data is collected through a mirroring port on the switch. Traces from two out of the four RTUs are used in previous work [24] and available to us. For the sake of consistency, we follow the naming scheme of the RTUs in the previous work and name the traffic as SmallTB-RTU1 and SmallTB-RTU4.
- **VirtualTB:** VirtualTB dataset is collected in a virtual testbed developed within the RICS project [1]. The testbed consists of an office network and a SCADA network. The setup of SCADA network contains some twenty substations, two SCADA servers, and a virtual WAN (Wide Area Network) with 15 nodes connecting the control room and the substations. The data is collected at the communication gateway to the WAN on the main SCADA server. There are no network delays or traffic congestion in this virtual network. Traces of one substation with an emulated RTU is used in earlier work [19] and available to us.
- **Real-RTUx:** Real-RTUx dataset is collected from a real power facility. The SCADA network contains several RTUs communicating with the SCADA server with different protocols. Among them, there are two RTUs that communicate through IEC-104 which are included in earlier work [20] and this study. The traffic is collected by the utility personnel running our data collection software in their operation site, here named Real-RTUA and Real-RTUB.

To perform our timing-based characterization, we need to transform the collected PCAP traces into desired formats: *event sequence* and *time series* of flows. The process includes the following steps. (1) It starts by identifying spontaneous packets with COT = SPONT. (2) For all the spontaneous traffic, the process separates them into unique flows, where a flow is defined by the tuple $RTU (SrcIP), IOA, TID$ . Note that a packet may contain multiple information objects and thus multiple IOA but only one TID as stated in Sect. 3. (3) The next step in preprocessing that forms an event sequence for each flow and records the PCAP timestamps as event arrival times for timing analysis. (4) Finally, the process transforms each event sequence to time series by calculating the number of events per some configurable interval of time.

No matter in which format, we split the events per flow into 10 parts, use the first part for learning and the remaining nine parts to evaluate the stability of the attributes. Table 1 shows an overview of the studied datasets with the associated throughput for each RTU. The TID column lists instructions found in the traffic from each RTU, and the last two columns present the number of flows found and used. In the previous works, the flows with low event rates were not included to avoid biased learning results. This study too excludes the flows with an event rate of fewer than 0.3 events per hour since these flows contain only sporadic events that apparently show very different attributes.

**Table 1.** Overview of time series obtained from the datasets.

Dataset	Duration	Throughput (#events/hr)	TID	# Flows	# Used Flows
SmallTB-RTU1	12 days	19182	M_ME_NA	4	4
SmallTB-RTU4	12 days	10712	M_ME_NA	3	3
VirtualTB	6 days	2433	M_ME_NA	15	12
Real-RTUA	30 days	13981	M_ME_NA	21	19
			M_DP_TB	8	0
			M_SP_TB	3	0
Real-RTUB	30 days	401	M_ME_NA	16	14

## Data Characterisation Methods

This section describes flow-based characteristics observed in the literature and the way we examine the characteristics in this paper.

### A Review of Potential Characteristics

This subsection briefly reviews three hypotheses about spontaneous traffic timing characteristics observed from earlier papers. [2, 15, 16, 19].

**Fig. 1.**
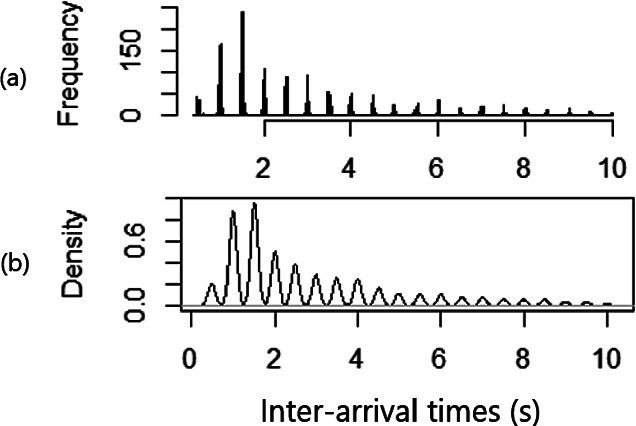
Distribution of inter-arrival times from a inter-arrival time sequence in the emulated VirtualTB dataset [19]: (a) Histogram for inter-arrival times $\delta _i \le 10$ s. (b) The smoothed version of the sequence, bandwidth = 0.008

- **Spiky Distribution.** Lin and Nadjm-Tehrani’s work [19] studies the timing predictability of the spontaneous events based on an assumption, namely that inter-arrival time distribution for events is spiky, without verifying it. A spiky distribution means the probability of some inter-arrival time to be present is higher than others as shown in Fig. 1(a).
- **Timing Predictability.** Timing predictability analysis addresses the research question: can we predict when the next spontaneous event will come by learning the historical timing data? In earlier work [19] it is shown that in 11 out of 14 tested data sequences, there exists evidence of sequential patterns. Hence, there is a hypothesis that historical data provides timing predictability even in spontaneous traffic.
- **Correlation.** There are a number of works that model sensor signals with clustering techniques based on correlations between sensors [2, 15, 16]. As stated in Sect. 3, sensor measurements of the processes and spontaneous events have a cause-effect relationship. The results indicate that sensors in SCADA systems are correlated. Therefore, we propose the correlation hypothesis that posits spontaneous events from different IOAs (i.e., connecting to different sensors) could be also correlated.

### A Review of Characterization Methods

This subsection describes the known methods that will be used to analyze the characteristics.

**Spiky Distribution.** Lin and Nadjm-Tehrani [19] propose an algorithm to learn the areas with high probability to have spikes as *legitimate areas*. The algorithm finds the relatively low point pairs on the smoothed curves of histogram as shown in Fig. 1(b), where the smoothing is done by the kernel density estimation method with a bandwidth parameter that decides the smoothness level. The low point pairs are considered as the boundaries of legitimate areas.

**Timing Predictability.** Following application of the algorithm mentioned in the previous paragraph, the same work separates inter-arrival times into groups, with one spike per group. The method translates the numeric event inter-arrival time sequence observed in a PCAP file (e.g., 3.15, 3.17, 0.51, 0.48) into a symbolic sequence by replacing each numeric inter-arrival time with its group symbol in the symbolic alphabet (e.g., aabb). Then, using the symbolic sequences, the method builds a PST for each flow in the learning phase and tests the predictability of the learned PST. In the testing phase, the method runs with a sliding window over the symbolic sequence. With a given window size (6 symbols in the mentioned study), the method queries the built PST for the next element that is most likely to happen as its prediction.

The mentioned work evaluates the timing predictability with predication accuracy and Kappa statistics [17]. With the resulting confusion matrix, the observed prediction accuracy $P_0$ is defined as:

1 $$\begin{aligned} P_0 = \frac{\sum _{i=1}^{c}n_{ii}}{N} \end{aligned}$$

where N is the number of predictions performed in the testing phase, c is the number of possible symbols (i.e., number of rows/columns of the confusion matrix), and $n_{jk}$ is the number of times the symbol k (ground truth) is predicted as j. The expected prediction accuracy by a random observer is:

2 $$\begin{aligned} P_e =\sum _{i=1}^{c} (\frac{n_{i+}}{N}\times \frac{n_{+i}}{N}) \end{aligned}$$

where $n_{i+}$ is the total number of times the symbol *i* appears in the testing data and $n_{+i}$ is the total number of times any symbol is predicted as *i*. Kappa statistics is:

3 $$\begin{aligned} Kappa =\frac{P_0-P_e}{1-P_e} \end{aligned}$$

A random observer is a pseudo observer who randomly picks up a value from the learned probability distribution of inter-arrival times. Kappa statistics compares the observed accuracy and expected accuracy. If our prediction model is similar to a random observer, the Kappa value will be around 0. On the other hand, if our prediction model and the testing data contains clear sequential patterns, the Kappa value will be close to 1.

**Correlation.** Spearman correlation coefficient ($\rho$) is a measure of the monotonic relationship of two time series. For any two time series $X^p=x-i^p,\dots ,x_m^p$ and $X^q=x_i^q,\dots ,x_m^q$, we have ranked time series $R(X^p)=R(x_1^p),\dots , R(x_m^p)$ and $R(X^q)=R(x_1^q),\dots , R(x_m^q)$, where the numeric values are replaced by their rank in the sorting. Then, the Spearman correlation coefficient is:

4 $$\begin{aligned} \rho _{pq} =\frac{COV(R(X^p), R(X^q))}{\sigma _{R(X^p)} \sigma _{R(X^q)}} \end{aligned}$$

where $COV(R(X^p),R(X^q))$ denotes the covariance of the ranked time series and $\sigma _{R(X^p)}$ and $\sigma _{R(X^q)}$ are the standard deviations.

The correlation coefficient values are between −1 and 1. The values close to 1 or −1 indicate a strong relation between the two time series in the same or opposite direction, and values close to 0 indicate a low association between time series.

### Methods and Parameter Choices for the Comparative Analysis

The comparative analysis in this paper aims to not only understand whether the above characteristics exist in the three datasets from Sect. 4 but also how persistent they are. This subsection elaborates the workflows and parameter choices for the comparative analysis.

**Spiky Distribution.** The analysis first illustrates and categorizes the Probability Density Function (PDF) of inter-arrival times. Then, it tests whether the characteristics are stable and persistent. In this paper we will learn the legitimate areas with a high probability to have spikes as shown in Sect. 5.2. The major difference between the implementation in this paper and the earlier work is the limitation of maximum number of spikes. Our implementation can find as many spikes as possible while the previous work has a limit on number of spikes set as 12, which means only the twelve largest spikes can be modeled.

Further, we test if the learned results remain in the testing part of the data using the metric of *unknown data rate*. The unknown data rate (UDR) is defined as:

5 $$\begin{aligned} UDR = \frac{n_x}{N} \end{aligned}$$

where $n_x$ denotes number of observations in the testing data that do not locate in any of the learned legitimate areas, and *N* denotes number of observations in the testing data. Thus, the lower the UDR, the higher the degree of stability of the spiky distribution.

**Timing Predictability.** The timing predictability comparative analysis applies the proposed method in Sect. 5.2 to all three datasets from Sect. 4. There are two different enhancements compared to the earlier work. First, based on the results of spiky distribution analysis, we can get as many symbolic alphabets as possible from an inter-arrival time sequence. This change makes the PST sequence model more accurate. Compared to the earlier work, the inter-arrival times located in the spikes that are smaller than the twelfth largest spike won’t be bundled together. Second, this study uses one-tenth of the datasets as learning data, which contains more observations than the 2-hour (short) learning data in the earlier work. This change enables the PST model to discover longer sequential patterns if there are any.

**Correlation.** We calculate how many pairs of time series are significantly correlated by computing p-values for null-hypothesis $H_0: \rho _{pq}=0$, and compare the correlation rates between different datasets. The bin size of the time series in this study is 1 min. With the resulting correlation rate of flows, we further examine how the spontaneous traffic flows are correlated with each other using dendrograms and if there’s any change on the dendrograms for learning period and testing period.

## Observations and Discussions

This section summarises the results of our comparisons between the emulated and real data sets, using the above hypotheses and applied methods.

### Spiky Distribution

**Fig. 2.**
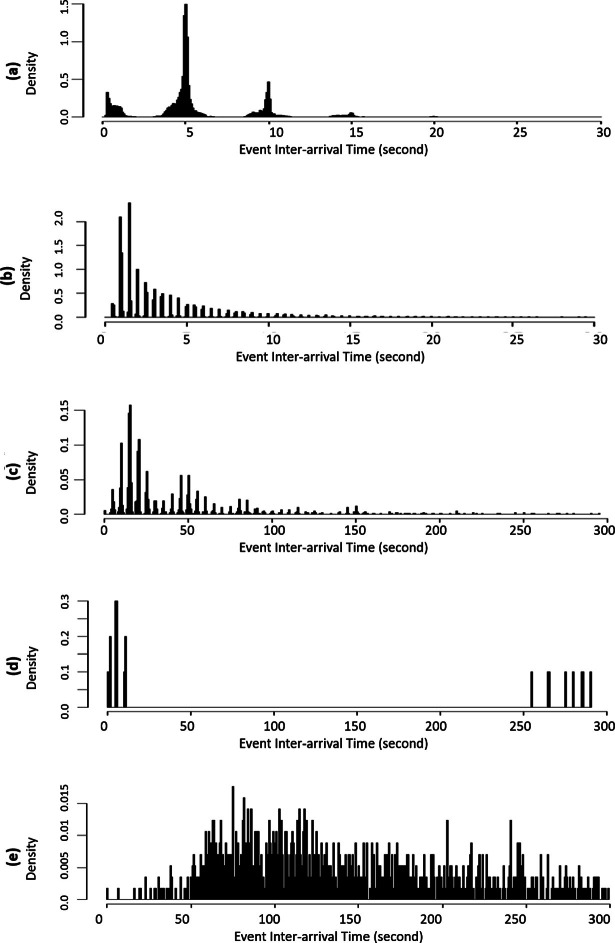
Common patterns in the PDF of event inter-arrival times. (a) A centered pattern from VirtualTB, IOA 10091 (b) A long-tail pattern from SmallTB-RTU4, IOA 2 (c) A multi-center pattern from VirtualTB, IOA 10002. (d) A dispersed pattern from VirtualTB, IOA 10010. (e) No clear pattern from Real-RTUB, IOA 3018.

A few common patterns appear in the PDFs of event inter-arrival times for each flow from the emulated datasets. These patterns contain multiple spikes with different heights and weights that are distributed as different shapes of curves. Figure 2(a) presents a centered pattern. The pattern contains one major spike and a few minor spikes located around it. Figure 2(b) is a long-tail pattern. The spikes are distributed with a long-tail. Figure 2(c) presents the multiple centered pattern in a long-tail distribution. Figure 2(d) presents spikes in a dispersed unknown distribution.

All the PDFs presented in emulated datasets show roughly equal spacing between the spikes and all the flows from emulated datasets have a constant size of gaps between spikes. The gap size between spikes in emulated datasets is the update rate at which the emulated RTUs update the information of simulated processes. If the value of monitored points changes or exceeds a predefined range when an RTU updates the information, the RTU sends a spontaneous event. VirtualTB has a gap size of 5 s and SmallTB has a gap size of 0.5 s.

As expected, most of the flows from the real traffic present spiky inter-arrival time distributions and equal spacing between the spikes. However, not all of the flows have the same gap size even if they are from the same RTU. The real data has the lowest gap size of 0.625 s and the largest gap size of 7.5 s. Moreover, in this dataset, traffic from different RTUs exhibit very different timing characteristics. Table 2 presents the inter-arrival time distribution type and UDR in column *Spiky Distribution*. Most of the flows issued by RTUA present centered patterns, whereas, in 10 out of 14 flows issued by RTUB, we did not find clear spikes as shown in Fig. 2(e). The flows without spikes have relatively low event rates (around 20 events per hour). We speculate that the real system monitors the processes with different granularities. Some are updated more often while some are not regularly updated.

The flows with a resulting unknown data rate (see Eq. 5) below 3$\%$ are highlighted in gray. Except for the flows that have less than 23 events per hour, all the flows with spiky inter-arrival time distributions exhibit a low UDR, which means the learned characteristics are stable and persistent within the data collection period. After a manual examination of the flows having low event rate and showing higher UDR, we observe that there are not enough elements in the learning period for the used algorithm to properly estimate the legitimate areas.

**Table 2.**
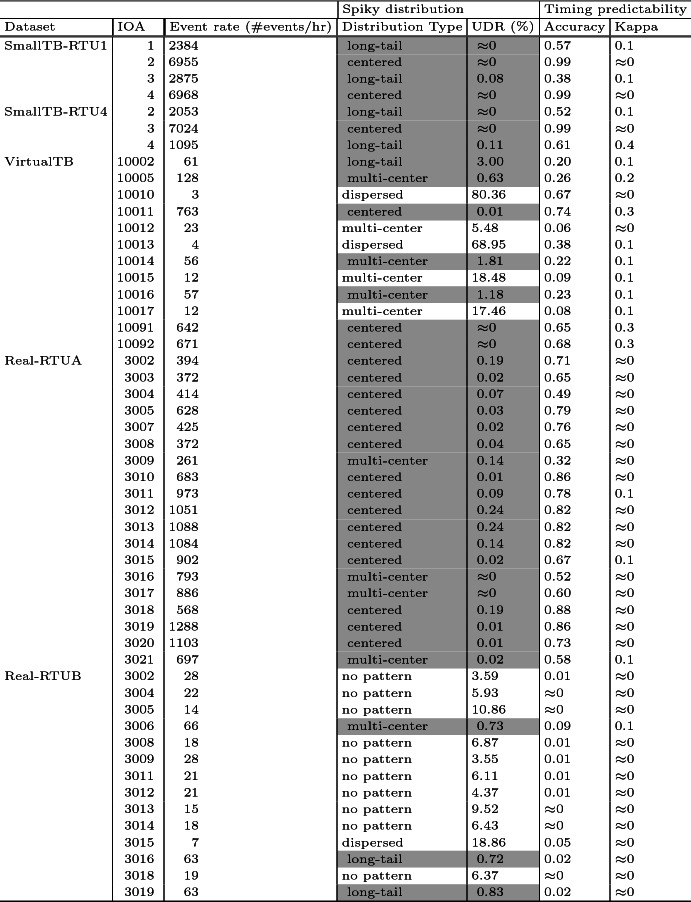
Analysis results for spiky distribution and timing predictability hypotheses. UDR stands for unknown data rate (Eq. 5).

### Timing Predictability

The timing predictability analysis results are presented in Table 2, the last two columns.

There are a few insights obtained in this analysis. First, as discovered in the earlier work [19], there are some flows in the emulated datasets that show evidence of the existence of sequential patterns. In 14 out of the 19 flows, we get a Kappa value that agrees on the existence of sequential patterns^1^ (i.e., Kappa is not around 0). Among them, 9 Kappa values show slight agreement (Kappa values around 0.1) and 4 show medium agreement (Kappa values 0.3–0.4). However, most of the flows from the Real-RTUx datasets have a Kappa value around 0 and only four flows have a slight agreement on the existence of sequential patterns. We speculate that the underlying sequences found in the emulated datasets could be generated by the repeated workflow of the process simulation programs.

Second, a first look at prediction accuracy may provide an impression that real data have lower accuracy. However, if we only look into the flows containing sufficient elements for learning (i.e., rows highlighted in gray), prediction accuracy is more related to distribution type rather than the type of dataset. Among all the highlighted flows, centered patterns give better accuracy in predictability irrespective of evidence of sequential patterns or not. Most of the flows of centered distribution type have high accuracy and a low Kappa value because most of the intervals fall into the major spike^2^. Long-tail patterns have barely predictability when the Kappa value is close to 0^3^, whereas they show higher prediction accuracy when there exists evidence of sequential patterns^4^. Multi-centered patterns have higher prediction accuracy when the distribution is closer to a centered distribution, i.e. most of the intervals fall into a few major spikes.

Third, compared with earlier work, our analysis gets improved accuracy for some flows from the emulated datasets^5^ due to the choice of learning parameters. The changes of the parameters include higher bandwidth for the kernel density estimation, extended learning phase, and unlimited number of symbols for the PSTs as described in Sect. 5.3.

### Correlation

**Fig. 3.**
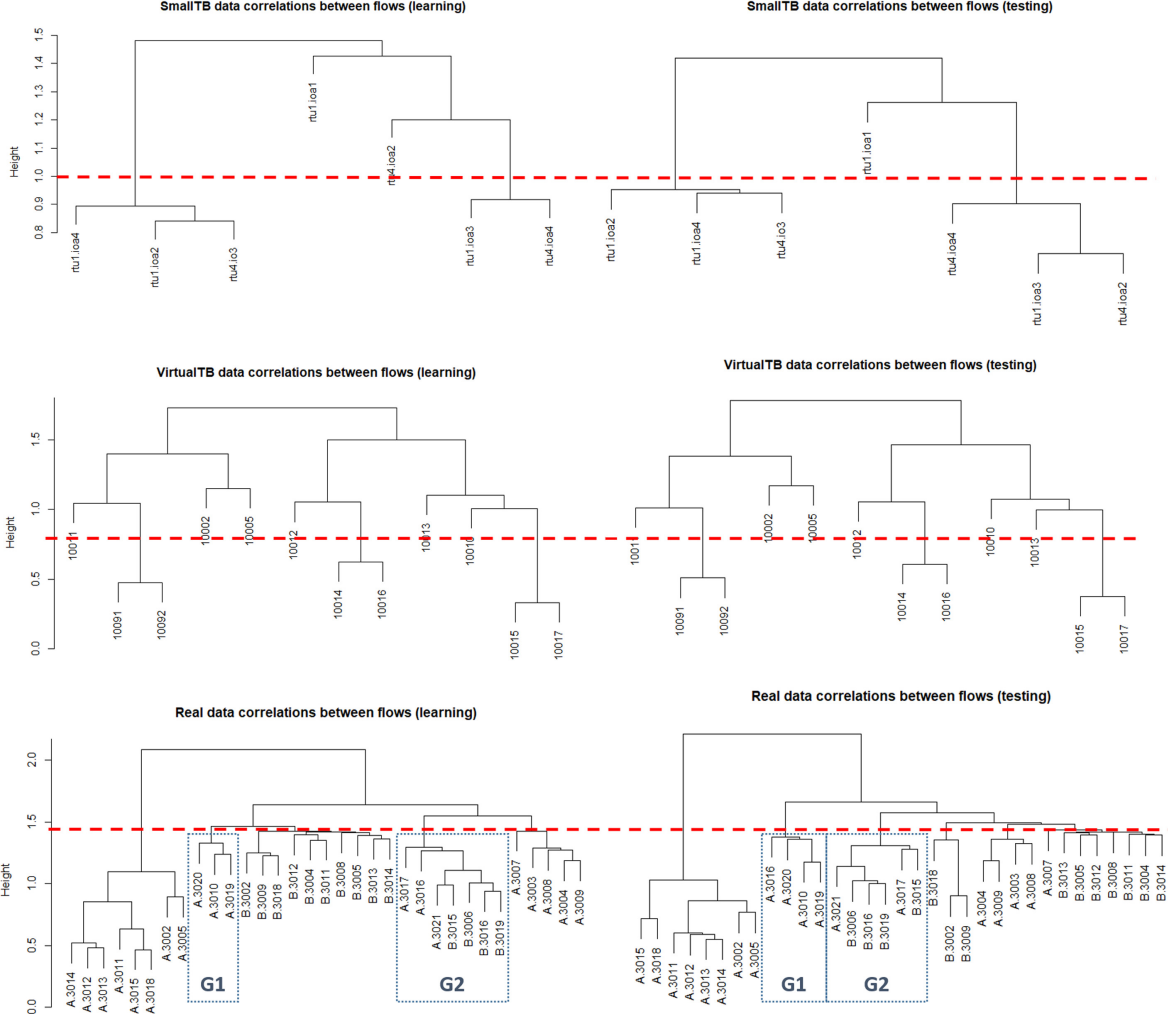
Correlation dendrograms for learning and testing period. Top: the small emulated network, Middle: the RICS-el virtual network, Bottom: the real utility network.

With a p-value of 0.05, there are 86%, 89%, and 74% significantly correlated flows respectively within SmallTB, VirtualTB, and Real datasets. Figure 3 presents the dendrograms using Euclidean distance between observations/clusters based on the absolute correlation. They show the observations/clusters for SmallTB, VirtualTB, and Real datasets in learning (left side) and testing period (right side), respectively. The leaves are the flow IDs, the height stands for Euclidean distance and the dotted line is an example cut-off line that separates the flows into clusters. The dendrograms of VirtualTB dataset have the same structure for learning and testing data. That is, for every cut-off line in the learning dendrogram, one can find a corresponding cut-off line in the testing dendrogram that generates the same clustering results.

In the dendrograms for SmallTB and Real dataset, there are a few flows that jump from one group to another but the structure remains the same for most of the time. For example, the cut-off line for Real data generates 6 groups in both the learning and testing period. There are two highlighted groups G1 and G2 in both trees. Flow RTUA 3016 is included in G2 of the learning tree, but it moves to G1 of the testing tree in the testing period.

The results suggest that correlations between flows are complicated. A flow can be correlated with multiple flows and the magnitude of correlations between different flows may change from time to time. We speculate that the virtual testbed has fewer dynamical processes so that it exhibits overly stable relations between flows.

## Conclusions

Due to the secrecy nature of SCADA traffic, lack of openly available datasets for intrusion detection research has been an open question. Many research efforts on intrusion detection systems in SCADA networks are tested with emulated or simulated datasets. This study examines three hypotheses about IEC-104 spontaneous traffic attributes that were proposed or observed in previous work with a comparison between emulated and real datasets. The results show that emulated datasets are prone to simple and regular patterns.

In the spiky distribution analysis, the emulated datasets exhibit a unified update rate of information that shows up as a unique gap size between spikes in the whole system. The real datasets, on the other hand, exhibit a wide variety of gap sizes in a system. Some of the flows even do not present a spiky inter-arrival time distribution.

In the predictability analysis, the emulated datasets exhibit evidence of underlying inter-arrival time sequences that make the timing of the next event predictable. However, the real dataset suggests little evidence of underlying sequences.

In the correlation analysis, both the emulated and real datasets indicate that traffic flows are intricately correlated. However, the correlations between flows seem to be less dynamic in emulated datasets.

The study of differences between emulated and real datasets ought to be a precondition for intrusion detection research, especially learning-based anomaly detection systems. The results in this paper show that traffic attributes that exist in emulated datasets may be not valid in real datasets. Therefore, it’s crucial to select explainable features for anomaly detection systems when only emulated datasets are available for learning and testing. The simpler and more regular attributes can lead to overestimation of performance as well. This indicates room for improvement of emulated datasets, such as more detailed and complicated system configurations or adding random events to the process simulators.

One obvious future work is to find more attributes from different real datasets and a systematic approach to generate realistic synthetic datasets. The results in this study suggest the need to characterize the uncertainty of the selected features. Another way is to make sanitized real datasets openly available by applying traffic anonymization methods.

## Footnotes

1 SmallTB-RTU1 IOA: 1, 3, SmallTB-RTU4 IOA: 2, 4, VirtualTB IOA: 10002, 10005, 10011, 10013 10014, 10015, 10016, 10017, 10091, 10092.

2 SmallTB-RTU1 IOA: 2, 4, SmallTB-RTU4 IOA: 3, Real-RTUA IOA: 3002, 3003, 3004, 3005, 3007, 3008, 3010, 3011, 3012, 3013, 3014, 3015, 3018, 3019, 3020.

3 Real-RTUB IOA: 3016, 3019.

4 SmallTB-RTU1 IOA: 1, 3, SmallTB-RTU4 IOA: 2, 4, VirtualTB IOA: 10002.

5 SmallTB-RTU1 IOA: 2, 4 and SmallTB-RTU4 IOA: 3.
